# Use of OTC-drugs prior to Hospitalization

**DOI:** 10.1186/1757-7241-21-S2-A51

**Published:** 2013-09-09

**Authors:** Magnus Pedersen, Mikkel Brabrand

**Affiliations:** 1 Sydvestjysk Sygehus, Esbjerg, Denmark

## Background

Use of over the counter (OTC)-drugs is increasing and as it is poorly registered, this can lead to complications. The most commonly used OTC-drugs are analgesics and use is highest among elderly. Our study investigates the use of OTC-drugs 24 hours prior to hospitalization as well as the effect of the drugs.

## Methods

The junior physicians on call interviewed all patients admitted to the medical admission unit at Sydvestjysk Sygehus in Esbjerg on the use of OTC-drugs, using a modified chart template designed for the purpose. All adult patients aged 15 and older admitted over a two week period in August 2012 were included. The patients were asked about the drugs taken, dosage, indication and effect. OTC-drugs where categorised based on ATC-codes.

## Results

From a total of 349 admissions 188 usable chart templates were registered (54%) and information on OTC usage was registered on 165 of these (88%). The patients were elderly (median: 70 years) and 43 reported an intake of OTC-drugs (26%). A total of 22 different OTC-drugs had been consumed with analgesics being the most widely used (74%). The majority of patients had taken the drugs on a relevant indication (88%), the most common indication being pain. Half the patients had taken the drugs in a relevant dosage (51%). Sixty percent felt an effect of the intake and the majority on pain symptoms.

## Conclusion

Our findings reveal that one in four patients use OTC-drugs 24 hours prior to hospitalization. Most patients use OTC-drugs relevantly and half with a positive effect. The intake is poorly registered, and there is a need for more focus on the intake of OTC-drugs to avoid potential side-effects and medicine-interactions due to this increasing intake.

